# Supplementary figures and images for: The Arthropoda-specific Tramtrack group BTB protein domains use previously unknown interface to form hexamers
Source: eLife. 2024 Sep 2;13:e96832. doi: 10.7554/eLife.96832 (PMC11426971; doi:10.7554/eLife.96832)

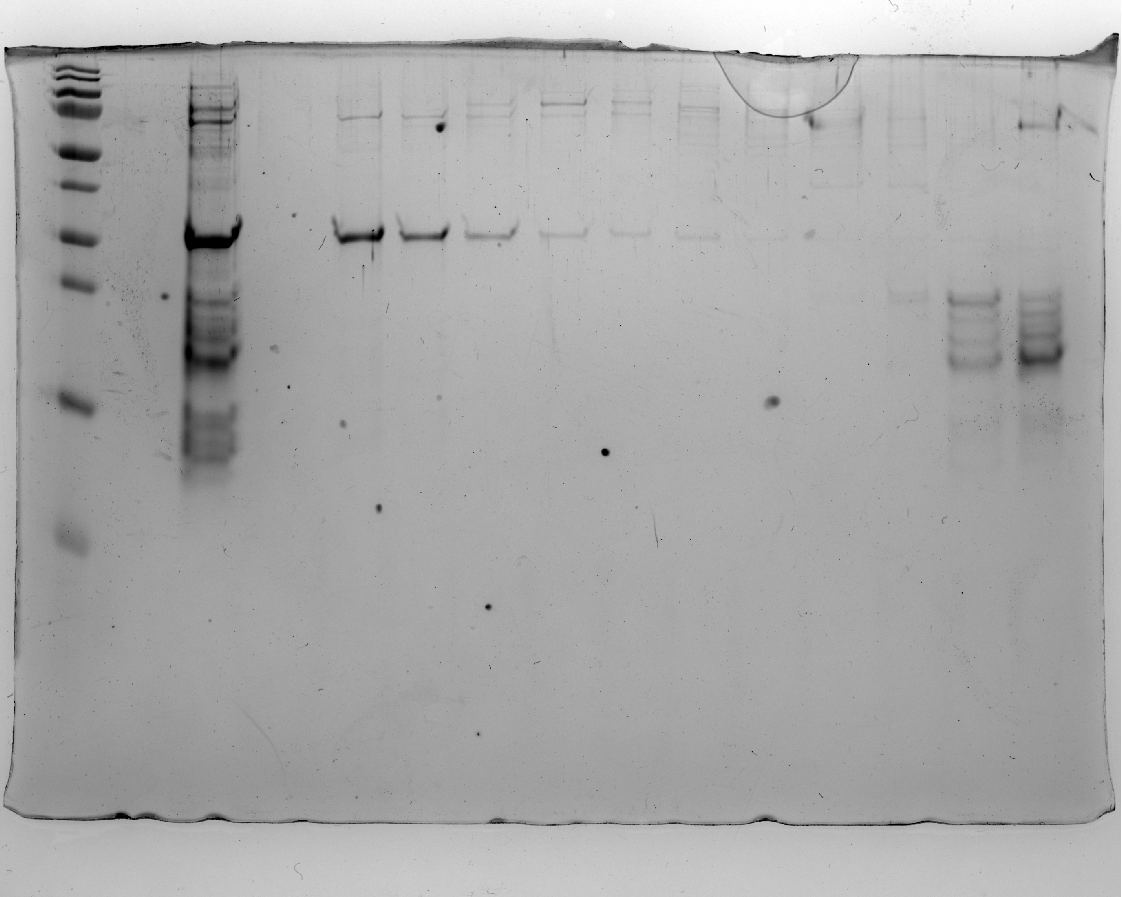

Supplement: Figure 4—figure supplement 4—source data 1. [file elife-96832-fig4-figsupp4-data1.zip › Figure 4—figure supplement 4_source_data/Figure-supplement-9-source_data1.tif]

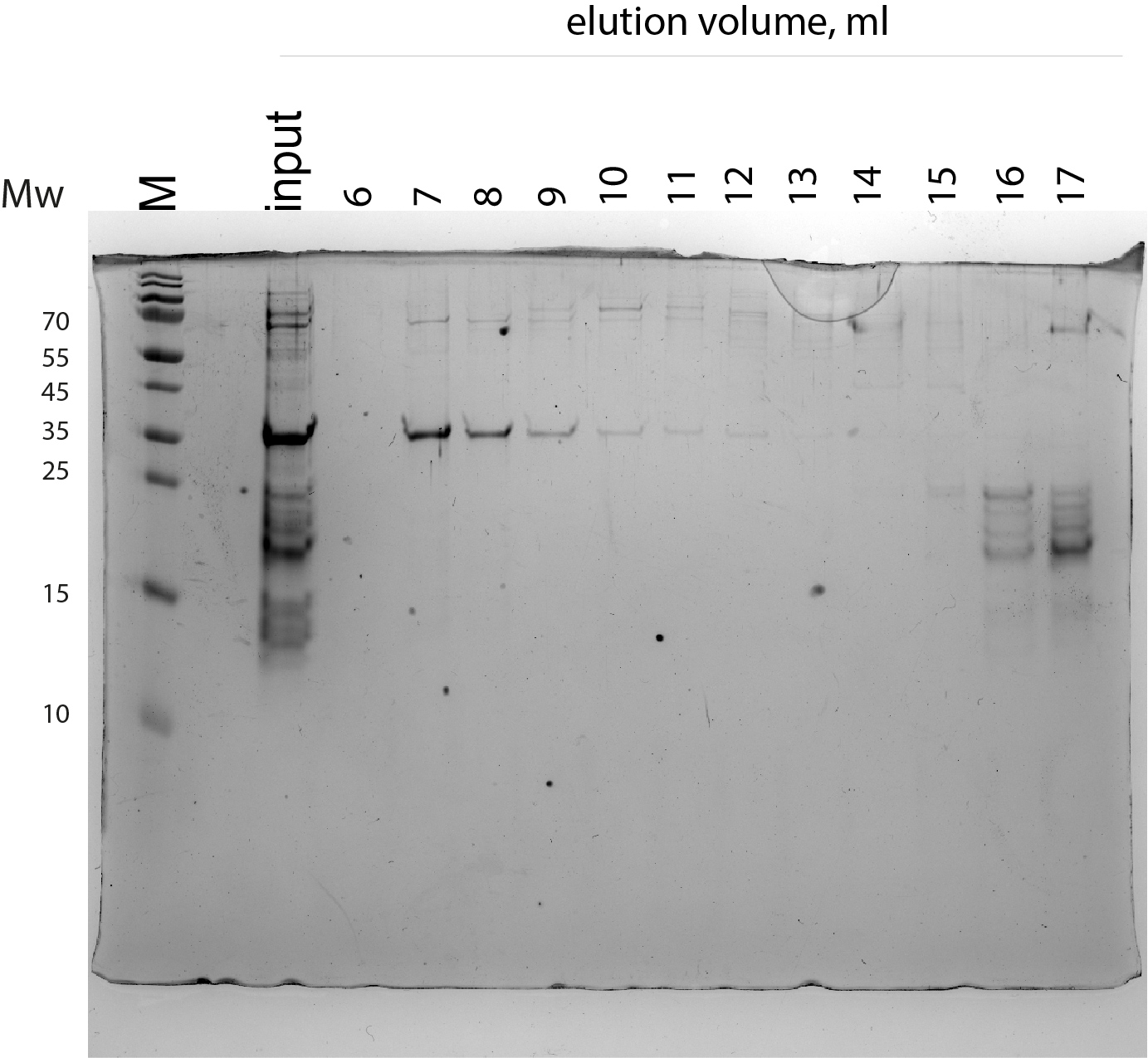

Supplement: Figure 4—figure supplement 4—source data 1. [file elife-96832-fig4-figsupp4-data1.zip › Figure 4—figure supplement 4_source_data/Figure-supplement-9-source_data2.jpg]

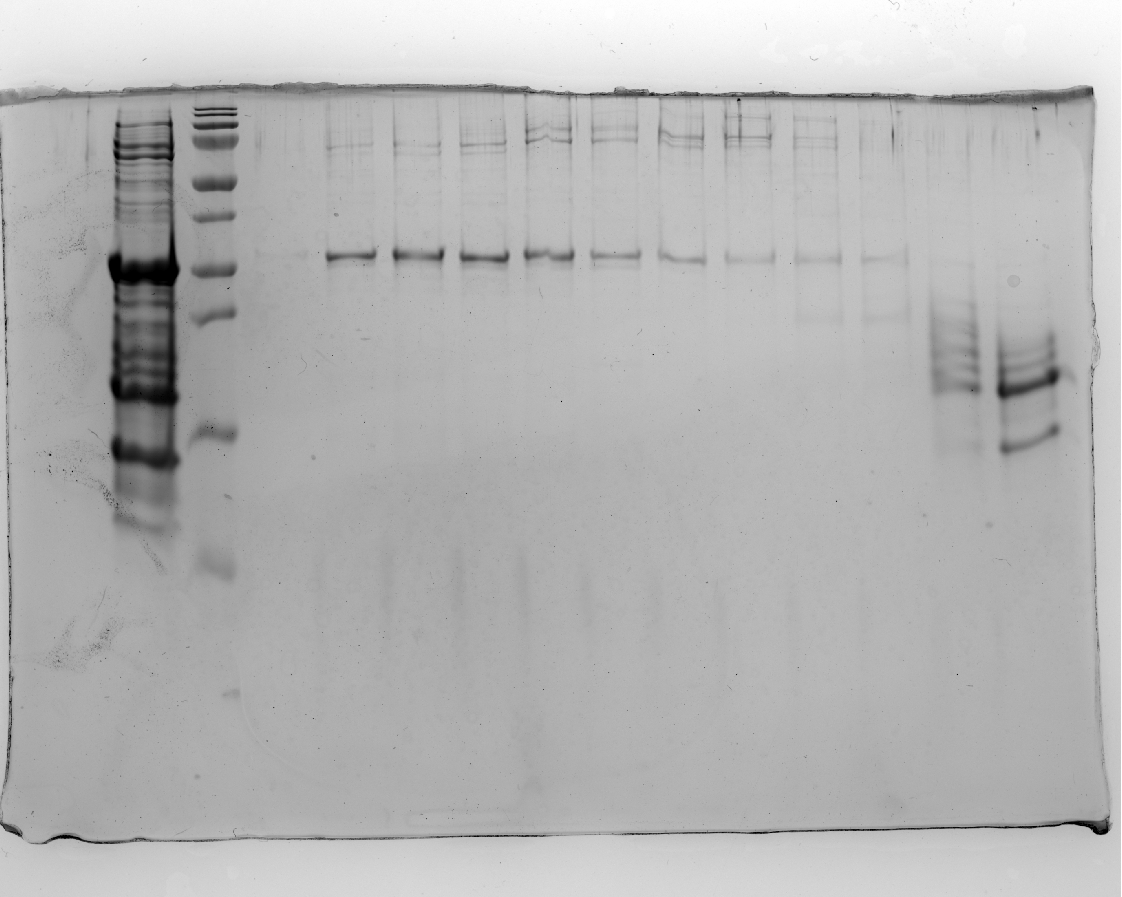

Supplement: Figure 4—figure supplement 5—source data 1. [file elife-96832-fig4-figsupp5-data1.zip › Figure 4—figure supplement 5_source_data/Figure-supplement-10-source_data1.tif]

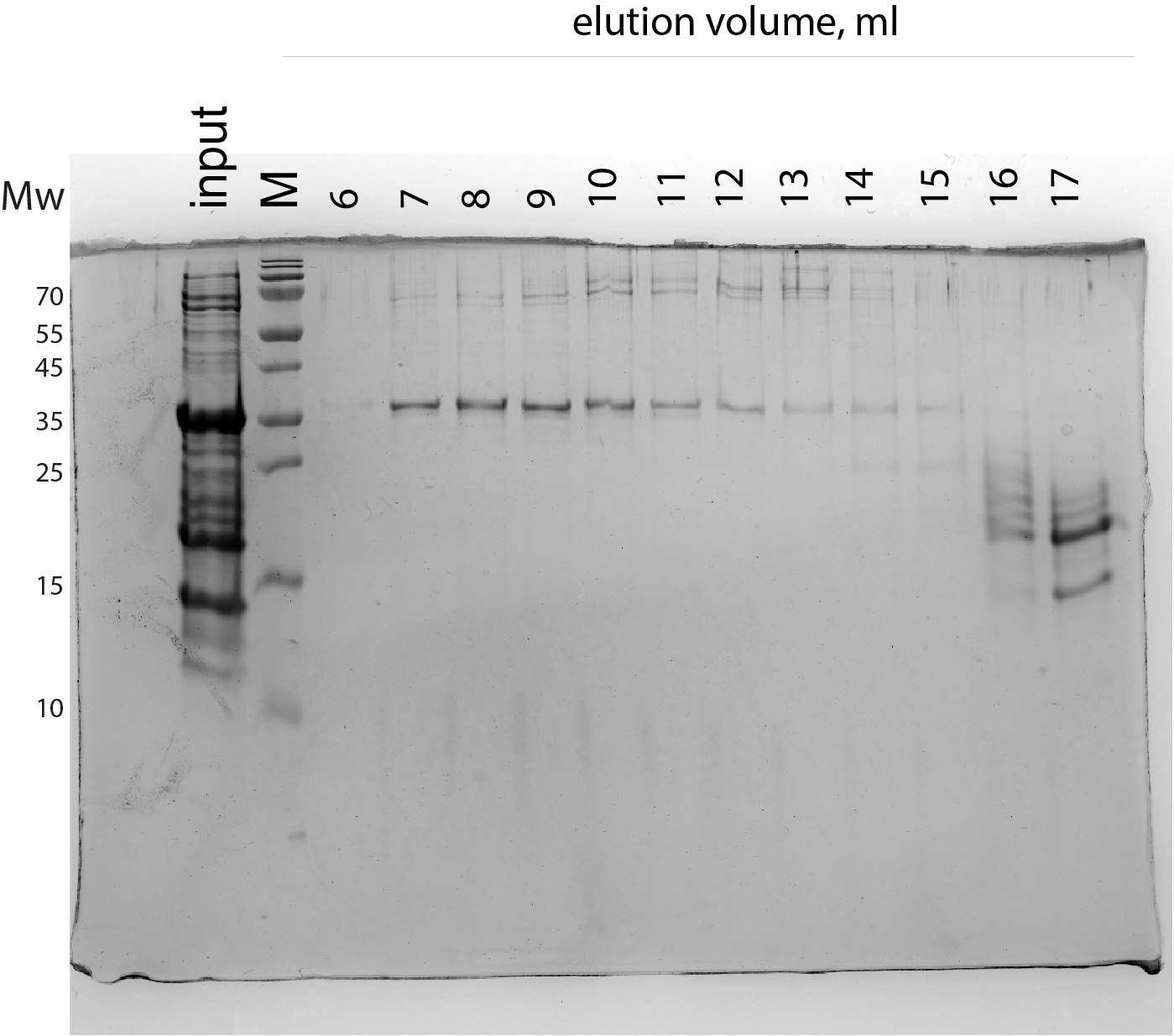

Supplement: Figure 4—figure supplement 5—source data 1. [file elife-96832-fig4-figsupp5-data1.zip › Figure 4—figure supplement 5_source_data/Figure-supplement-10-source_data2.jpg]

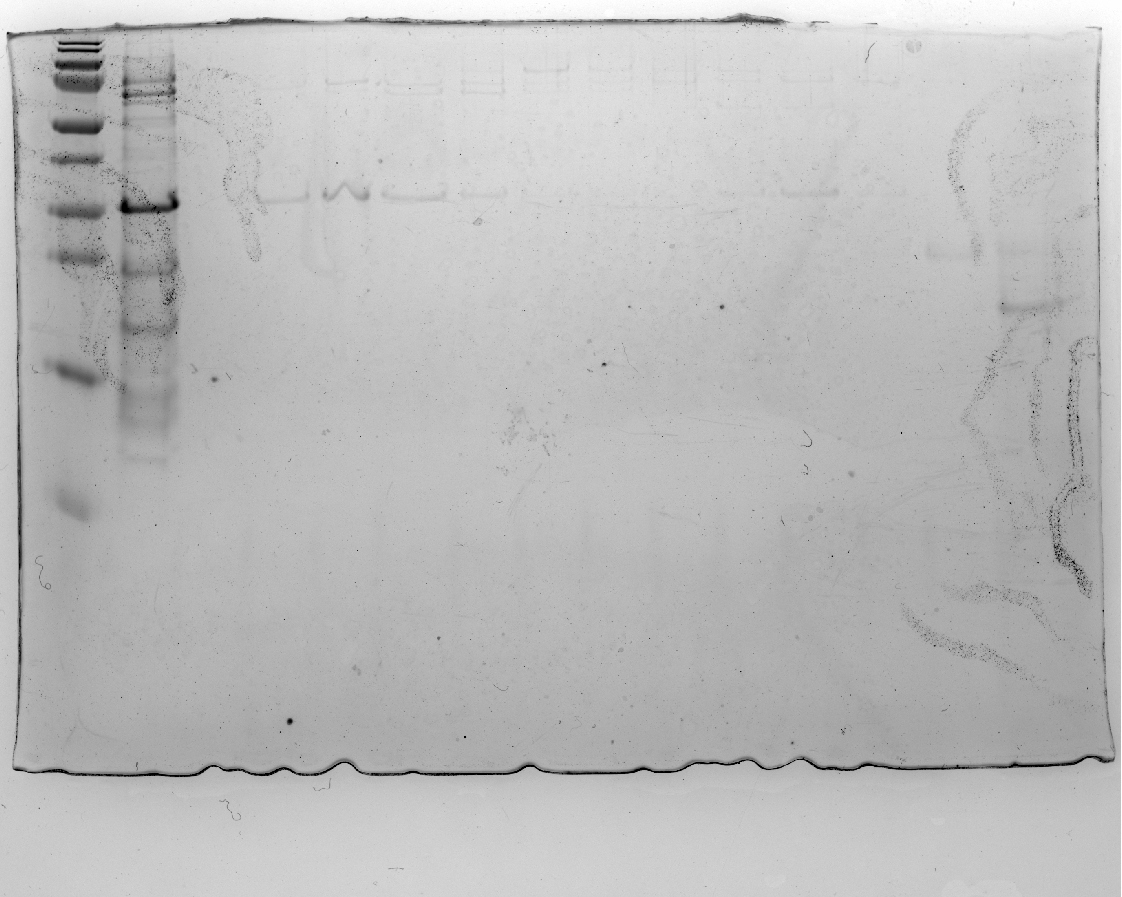

Supplement: Figure 4—figure supplement 6—source data 1. [file elife-96832-fig4-figsupp6-data1.zip › Figure 4—figure supplement 6_source_data/Figure-supplement-11-source_data1.tif]

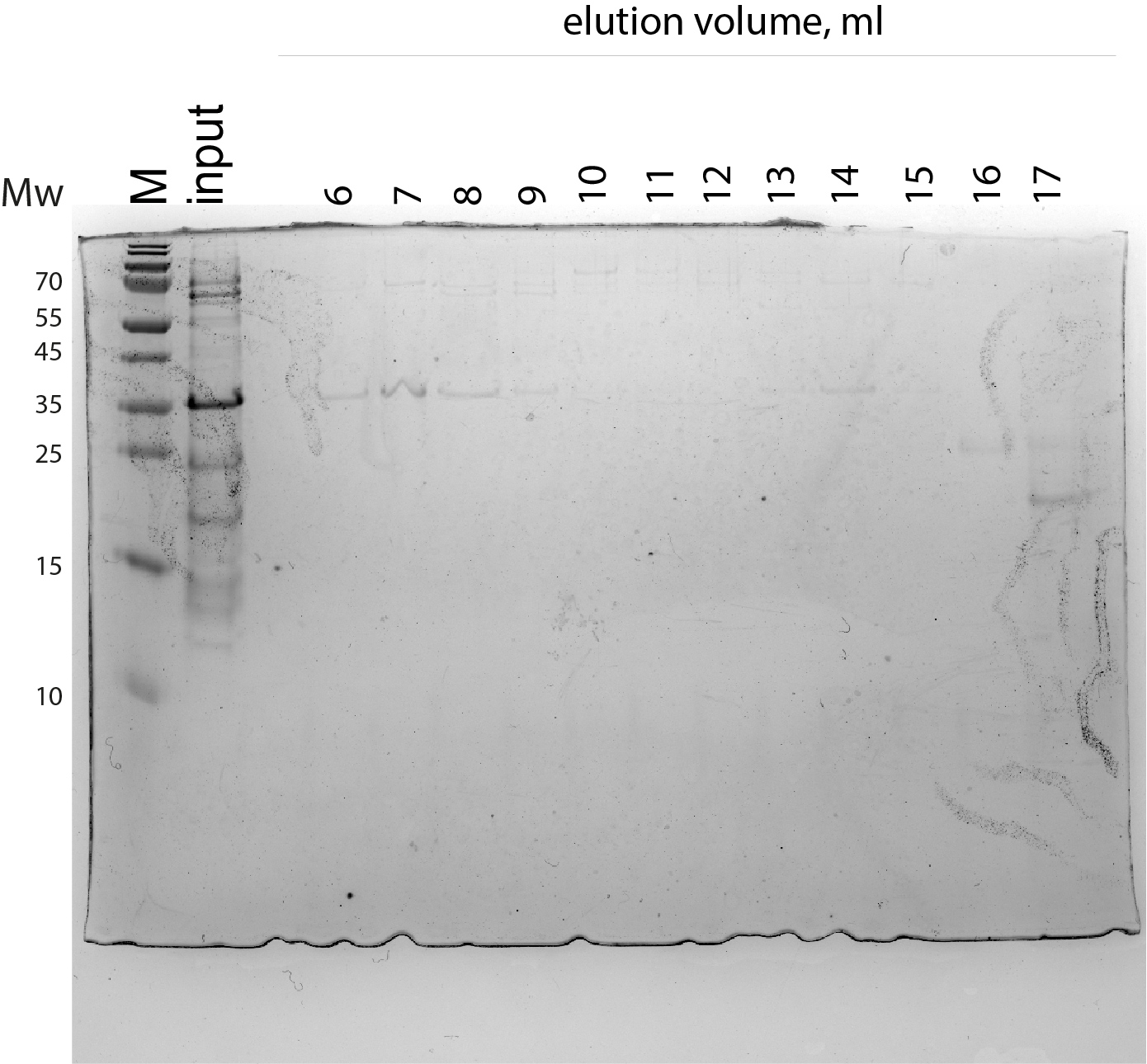

Supplement: Figure 4—figure supplement 6—source data 1. [file elife-96832-fig4-figsupp6-data1.zip › Figure 4—figure supplement 6_source_data/Figure-supplement-11-source_data2.jpg]
