# Supplementary material for: The Arthropoda-specific Tramtrack group BTB protein domains use previously unknown interface to form hexamers
Source: eLife. 2024 Sep 2;13:e96832. doi: 10.7554/eLife.96832 (PMC11426971; doi:10.7554/eLife.96832)
Supplement: Supplementary file 1. [file elife-96832-supp1.docx]

**Supplementary file 1. Cryo-EM statistics. Data collection, processing, model refinement and validation statistics.**

| **Data collection and processing**  Magnification 165,000 |
| --- |
| Voltage(kV) 300 |
| Electron 60 |
| exposure(eˉ/Å²) |
| Energy filter slit — width(eV) |
| Defocus range(μm) -0.8 to -2.2 |
| Pixel size(Å) 0.729 |
| Symmetry imposed C1 |
| Movies(no.) 9,765 |
| Initial particle 480,721 images(no.) |
| Final particle 197,562 |
| images(no.) |
| Map resolution(Å) 3.3  FSC threshold. 0.143 |
|  |
| **Refinement** |
| Initial model used AlphaFold |
|  |
| Model composition |
| Non-hydrogen 6118 |
| atoms |
|  |
| Protein residues 780 |
| R.m.s deviations  Bond lengths(Å) 0.005 |
| Bond angles(°) 0.553 |
| **Validation** |
| MolProbity score 1.80 |
| Clashscore 6.07 |
| Rotamer outliers(%) 0.58 |
| Ramachandran plot Favored(%) 98.57 |
| Allowed(%) 1.30 |
| Disallowed(%) 0.13 |
| Model to map CC 0.77/0.76/ |
| (mask, peaks, volume) 0.66 |
