# Supplementary material for: The Arthropoda-specific Tramtrack group BTB protein domains use previously unknown interface to form hexamers
Source: eLife. 2024 Sep 2;13:e96832. doi: 10.7554/eLife.96832 (PMC11426971; doi:10.7554/eLife.96832)
Supplement: Supplementary file 2. [file elife-96832-supp2.docx]

**Supplementary file 2**. The ability of GAF, Mod(mdg4), Chinmo, CG8924 and LOLA BTB domains to interact with other TTK-type BTB domains in yeast two-hybrid assay. BCL6 is heterologous BTB domain of human BCL6 protein. GAF was tested both as AD and BD fusions, whereas Mod(mdg4), Chinmo, CG8924 and LOLA were tested only as BD fusions. Interactions with CG32121 were tested only for AD fusions due to strong self-activation properties. AD stands for Activation Domain, BD – for DNA-Binding Domain of GAL4 protein. + or - denotes the ability of yeasts to grow on the media without histidine. Yeast assay plates are shown in Figure 2—figure supplement 1–6.

|  |  | AD | | |  |
| --- | --- | --- | --- | --- | --- |
|  |  | GAF BTB | - | dimerization control |  |
| BD | GAF | + | − | + |  |
|  | Abrupt | + | − | + |  |
|  | CG3726 | + | − | + |  |
|  | CG12236 | + | − | + |  |
|  | BTB VII | + | − | + |  |
|  | bab2 | + | − | + |  |
|  | bab1 | + | − | + |  |
|  | Ribbon | + | − | + |  |
|  | mod | + | − | + |  |
|  | lola | + | − | + |  |
|  | ttk | + | − | + |  |
|  | Psq | + | − | + |  |
|  | BCL6 | − | − | + |  |
|  | Batman | + | − | + |  |
|  | CG6118 | + | − | + |  |
|  | CG15812 | − | − | + |  |
|  | CG34376 | + | − | + |  |
|  | Fruitless | − | − | + |  |
|  | TKR | + | − | + |  |
|  | CG8924 | + | − | + |  |
|  | mamo | + | − | + |  |
|  | BRC | + | − | + |  |
|  | Chinmo | − | − | + |  |
|  | CG6765 | + | − | + |  |
|  | - | − | − |  |  |
|  |  |  |  |  |  |

|  |  | BD | | | | | |
| --- | --- | --- | --- | --- | --- | --- | --- |
|  |  | mod(mdg4) BTB | LOLA BTB | GAF BTB | CG8924 BTB | Chinmo BTB | - |
| AD | mod(mdg4) | + | − | + | − | − | − |
|  | CG32121 | + * | + | + | − | − | − |
|  | Abrupt | − | − | + | − | + | − |
|  | CG3726 | + | + | + | + | + | − |
|  | CG12236 | − | − | − | − | + | − |
|  | BTB VII | + | + | + | − | − | − |
|  | bab2 | + | − | + | + | + | − |
|  | bab1 | + | − | + | + | + | − |
|  | Ribbon | + | + | − | + | + | − |
|  | GAF | + | + | + | − | − | − |
|  | lola | − | + | + | − | − | − |
|  | ttk | − | + | + | + | + | − |
|  | Psq | − | − | + | + | + | − |
|  | BCL6 | − | − | − | − | − | − |
|  | Batman | + | − | + | + | + | − |
|  | CG6118 | + | − | + | + | + | − |
|  | CG15812 | + | − | + | + | + | − |
|  | CG34376 | + | − | + | + | − | − |
|  | Fruitless | + | + | − | + | + | − |
|  | TKR | + | + | + | + | + | − |
|  | CG8924 | − | − | + | + | + | − |
|  | mamo | − | + | + | + | + | − |
|  | BRC | + | − | + | − | + | − |
|  | Chinmo | − | − | − | − | + | − |
|  | CG6765 | − | − | + | − | + | − |
|  | - | − | − | − | − | − | − |
