# Supplementary material for: The Arthropoda-specific Tramtrack group BTB protein domains use previously unknown interface to form hexamers
Source: eLife. 2024 Sep 2;13:e96832. doi: 10.7554/eLife.96832 (PMC11426971; doi:10.7554/eLife.96832)
Supplement: Supplementary file 3. [file elife-96832-supp3.docx]

**Supplementary file 3.** Pairwise level of similarity between GAF and Mod(mdg4) BTB domains with other TTK-type domains. The level of homology was calculated as ratio of identical and conserved (according to CLUSTAL definitions) residues to the total number of residues.

|  | GAF | mod(mdg4) |
| --- | --- | --- |
| GAF | 100 | 35 |
| CG32121 | 36 | 39 |
| Abrupt | 47 | 40 |
| CG3726 | 35 | 40 |
| CG12236 | 35 | 42 |
| BTB VII | 40 | 47 |
| bab2 | 37 | 43 |
| bab1 | 42 | 42 |
| Ribbon | 34 | 35 |
| mod (mdg4) | 35 | 100 |
| lola | 38 | 45 |
| ttk | 38 | 47 |
| Psq | 38 | 37 |
| Batman | 39 | 44 |
| CG6118 | 33 | 47 |
| CG15812 | 27 | 25 |
| CG34376 | 39 | 45 |
| Fruitless | 31 | 51 |
| TKR | 37 | 36 |
| CG8924 | 33 | 37 |
| mamo | 38 | 44 |
| BRC | 46 | 43 |
| Chinmo | 32 | 40 |
| CG6765 | 27 | 32 |
