# Supplementary material for: The Arthropoda-specific Tramtrack group BTB protein domains use previously unknown interface to form hexamers
Source: eLife. 2024 Sep 2;13:e96832. doi: 10.7554/eLife.96832 (PMC11426971; doi:10.7554/eLife.96832)
Supplement: Supplementary file 4. [file elife-96832-supp4.docx]

**Supplementary file 4.** Testing of the interactions between the non-TTK BTB domains of C2H2 proteins. (*) – the growth was assessed in the presence of 3-aminotriazole due to strong self-activation properties. Yeast assay plates are shown in Figure 2—figure supplement 7–11.

|  | | AD | | | | |
| --- | --- | --- | --- | --- | --- | --- |
|  |  | CP190 | ken | CG6792 | CG15275 | - |
| BD | CP190 | **+** | − | − | − | − |
|  | ken | **-** | **+** | **-** | − | − |
|  | CG6792* | − | − | **+** | − | − |
|  | CG15275 * | − | − | − | **+** | − |
|  | - | − | − | − | − | − |
