# Supplementary material for: The Arthropoda-specific Tramtrack group BTB protein domains use previously unknown interface to form hexamers
Source: eLife. 2024 Sep 2;13:e96832. doi: 10.7554/eLife.96832 (PMC11426971; doi:10.7554/eLife.96832)
Supplement: Supplementary file 5. [file elife-96832-supp5.docx]

**Supplementary file 5.** The ability of non-ttk BTB domains of CP190, CG6792, CG15725 and Ken proteins to interact with other TTK-type BTB domains in yeast two-hybrid assay. Positive results from initial screen against AD-tagged baits were further studied in reciprocal experiment as BD-tagged (lower table). BCL6 is heterologous BTB domain of human BCL6 protein used as an additional control. Designations are the same as in Supplementary file 2. Yeast assay plates are shown in Figure 2—figure supplement 7–11.

|  |  |  | AD | | | | |  |
| --- | --- | --- | --- | --- | --- | --- | --- | --- |
|  |  | CP190 BTB | | Ken BTB | CG6792 BTB | CG15725  BTB | - |  |
| BD | mod(mdg4) | − | | − | − | − | − |  |
|  | CG32121 | −* | | −* | −* | −* | −* |  |
|  | Abrupt | − | | − | − | − | − |  |
|  | CG3726 | − | | − | − | − | − |  |
|  | CG12236 | − | | − | − | − | − |  |
|  | BTB VII | − | | − | − | − | − |  |
|  | bab2 | − | | − | − | − | − |  |
|  | bab1 | − | | − | − | − | − |  |
|  | Ribbon | + | | − | − | − | − |  |
|  | GAF | + | | − | − | − | − |  |
|  | lola | − | | − | − | − | − |  |
|  | ttk | − | | − | − | − | − |  |
|  | Psq | − | | − | − | − | − |  |
|  | BCL6 | − | | − | − | − | − |  |
|  | Batman | − | | + | − | − | − |  |
|  | CG6118 | − | | − | − | − | − |  |
|  | CG15812 | − | | − | − | − | − |  |
|  | CG34376 | − | | − | − | − | − |  |
|  | Fruitless | − | | − | − | − | − |  |
|  | TKR | − | | − | − | − | − |  |
|  | CG8924 | − | | − | − | − | − |  |
|  | mamo | − | | + | − | − | − |  |
|  | BRC | − | | − | − | − | − |  |
|  | Chinmo | − | | − | − | − | − |  |
|  | CG6765 | − | | − | − | − | − |  |
|  | - | − | | − | − | − | − |  |

|  | | BD | |
| --- | --- | --- | --- |
|  |  | CP190 BTB | - |
| AD | Ribbon | + | − |
|  | GAF | − | − |
|  | - | − | − |
