# Supplementary material for: The Arthropoda-specific Tramtrack group BTB protein domains use previously unknown interface to form hexamers
Source: eLife. 2024 Sep 2;13:e96832. doi: 10.7554/eLife.96832 (PMC11426971; doi:10.7554/eLife.96832)
Supplement: Supplementary file 6. [file elife-96832-supp6.docx]

**Supplementary file 6.** AlphaFold2-Multimer – modeled heteromeric interactions of 4 subunits of one BTB domain (column) with 2 subunits of another BTB (rows) for pairs with interaction confirmed in Y2H assay (+). Grey cells indicate pairs where formation of heteromer was predicted to use dimer-dimer interaction interface, white cells indicate pairs where preferential heterodimer formation was suggested.

|  |  | BD | | | |  |
| --- | --- | --- | --- | --- | --- | --- |
|  |  | LOLA BTB | CG8924 BTB | Chinmo BTB | mod(mdg4) BTB | GAF BTB |
| AD | mod(mdg4) | − | − | − | + | + |
|  | CG32121 | + | − | − | + | + |
|  | Abrupt | − | − | + | − | + |
|  | CG3726 | + | + | + | + | + |
|  | CG12236 | − | − | + | − | − |
|  | BTB VII | + | − | − | + | + |
|  | bab2 | − | + | + | + | + |
|  | bab1 | − | + | + | + | + |
|  | Ribbon | + | + | + | + | − |
|  | GAF | + | + | − | + | + |
|  | lola | + | − | − | − | + |
|  | ttk | + | + | + | − | + |
|  | Psq | − | + | + | − | + |
|  | BCL6 | − | − | − | − | − |
|  | Batman | − | + | + | + | + |
|  | CG6118 | − | + | + | + | + |
|  | CG15812 | − | + | + | + | + |
|  | CG34376 | − | + | − | + | + |
|  | Fruitless | + | + | + | + | − |
|  | TKR | + | + | + | + | + |
|  | CG8924 | − | + | + | − | + |
|  | mamo | + | + | + | − | + |
|  | BRC | − | − | + | + | + |
|  | Chinmo | − | − | + | − | − |
|  | CG6765 | − | − | + | − | + |
|  | - | − | − | − | − | − |
