# Supplementary material for: The Arthropoda-specific Tramtrack group BTB protein domains use previously unknown interface to form hexamers
Source: eLife. 2024 Sep 2;13:e96832. doi: 10.7554/eLife.96832 (PMC11426971; doi:10.7554/eLife.96832)
Supplement: Supplementary file 10. [file elife-96832-supp10.docx]

**Supplementary file 10**. Oligonucleotides used for cloning. Restriction enzyme sites are shown in small letters, the corresponding enzymes are noted. Nucleotide substitutions in mutagenic primers are also shown in small letters.

| **Name** | **Sequence** | **restriction enzyme** |
| --- | --- | --- |
| Bcl6 BTBdir | TAagatctAAAATGGCCTCGCCGG | BglII |
| Bcl6 BTBrev | TTAagcgctGGCCTTAATAAACTTCCG | *Eco47III* |
| GAGAdir | TAagatctACGATGTCGCTGCCAATG | *BglII* |
| GAGA 120 rev | ATggatccCGTCTGCGGTGCCAG | *BamHI* |
| batman_d | TTCTtcatgaTGTCGTCGGATC | *PagI* |
| batman_r | AATActcgagACCTTCCCGCTTTA | *Xho*I |
| psq_d | TACGccatggCAGCGGTTC | *NcoI* |
| psq_120r | CGTTctcgagGGTTTCGCAGA | *XhoI* |
| FRU_d | CAAggatccATGGACCAGCAATTCTGCTTG | *BamHI* |
| FRU_118r | GTActcgagATTGTTGTTATCTGTGAGACCAC | *XhoI* |
| LOLA_d | GAAggatccATGGATGACGATCAGCAG | *BamHI* |
| LOLA_120r | TCCctcgagAGTGCGATTGTCCGAAAGG | *XhoI* |
| BRC_d | ATCggatccATGGACGACACACAGCACTTC | *BamHI* |
| BRC_121r | ATGctcgagCTCTGCCTGCTGCTGCGTG | *XhoI* |
| BAB1_97d | ATCggatccAGCTCCCAGCAATTCTG | *BamHI* |
| BAB1_215r | AGCctcgagATGGGTCACATCCGCCAGAC | *XhoI* |
| BAB2_194d | ATCggatccGAGGGGCAGCAGTTCTGC | *BamHI* |
| BAB2_313r | TTCctcgagCCTGCCCGCACTGACCTC | *XhoI* |
| BTB VIId | CTCggatccATGTCCGTGCAGCAGTTC | *BamHI* |
| BTBVII-119r | TAActcgagttaCGTTGGCATCTCCGCTAGG | *XhoI* |
| 6765d | AACggatccATGGCCGCCGAAAACTATCAC | *BamHI* |
| 6765_133r | ATTctcgagTTAGGTGCGGCAGAGGCCAC | *XhoI* |
| ribbon_d | AAggatccATGGGCGGCCCAACGGCG | *BamHI* |
| ribbon_137r | TTAGCTGGCATGATTGAACTTCATC |  |
| chinmo_d | CTagatctATGGATCCGCAGCAGCAGTTC | *BglII* |
| chinmo121r | ttaTCCCGTTTCCGTGGACAGAC |  |
| 3726_d | GCggatccATGCTGCCGCAGCAGTAC | *BamHI* |
| 3726_125r | TTAGTCTTCGTCATCGCGCCAGG |  |
| mamo_d | ATCggatccATGGGCAGTGAGCACTACTG | *BamHI* |
| mamo_121r | AGctcgagTTACTGGTTCGTCATCTCGGCCAG | *XhoI* |
| 6118_306d | TTCggatccATCGATCAGTACCTGC | *BamHI* |
| 6118_419r | CAGgtcgacCTATGTGTGCAGCCCCTTG | *SalI* |
| CG15812d | CACggatccATGAATCATCTTAAGTGGATG | *BamHI* |
| CG15812_Br | TGGgtcgacTTAAAAGCTGATGGCAGATTTG | *SalI* |
| CG34376d | TCAggatccATGGACGACGAGTTTAAGC | *BamHI* |
| CG34376_Br | CGAgtcgacTTAATTGGTAGCCAAGCCCTTG | *SalI* |
| TTK_d | GTCggatccATGAAGATGGCATCTCAACG | *BamHI* |
| TTK_117r | ATCgtcgacGGTGAGGCCCTTGATGCG | *SalI* |
| CG15275d | attggatccATGTTCACCAATTGGCTGACTG | *BamHI* |
| CG15275_191r | atactcgagttaTTGAGCGCGCGCCAAAAATGAGCG | *XhoI* |
| CG6792d | GCTGgatccATGCTGCACTCACAGACAATGC | *BamHI* |
| CG6792_134r | CTTgtcgacttaCAAGGGCTGCAACTGGTAGAG | *SalI* |
| Ken1d | TCCggatccATGAAAGAGTTTCAAAGAATGTTG | *BamHI* |
| Ken147r | tatgtcgacttaGTACTGCTGCTTCCGCCTG | *SalI* |
| 6765_Y84K V86Kd | GCTGaagGTGaagCTGCCGCCGGATC |  |
| 6765_Y84K V86Kr | GCAGcttCACcttCAGCACTCCATTGGGAT |  |
| LOLA I70 I72Ad | CCCgccTTTgcaCTCAAGGATGTCAAGTAC |  |
| LOLA I70 I72Ar | CTTGAGtgcAAAggcGGGATGTTTGTCGTACTG |  |
| LOLA I70 I72Kd | CCCaagTTTaaaCTCAAGGATGTCAAGTAC |  |
| LOLA I70 I72Kr | CCTTGAGtttAAActtGGGATGTTTGTCGTACTG |  |
| MOD I71K F73Kd | CGCTaagGTAaagCTGAACAACGTCAGCCAC |  |
| MOD I71K F73Kr | GTTGTTCAGcttTACcttAGCGTGGGTGTTCGAC |  |
| MOD I71A F73Ad | GCTgccGTAgccCTGAACAACGTCAGCC |  |
| MOD I71A F73Ar | CAGggcTACggcAGCGTGGGTGTTCGAC |  |
| MOD_F73Ad | GCTATCGTAgcCCTGAACAACGTCAGCC |  |
| MOD_F73Ar | CGTTGTTCAGGgcTACGATAGCGTGGGTG |  |
| MOD_F73Pd | GCTATCGTAccCCTGAACAACGTCAGCC |  |
| MOD_F73Pr | CGTTGTTCAGGggTACGATAGCGTGGGTG |  |
| LOLA72Ad | CCCATCTTTgcACTCAAGGATGTCAAGTAC |  |
| LOLA72Ar | CTTGAGTgcAAAGATGGGATGTTTGTCGTACTG |  |
| LOL70P72Ad | CCCccCTTTgcACTCAAGGATGTCAAGTAC |  |
| LOL70P72Ar | CTTGAGTgcAAAGggGGGATGTTTGTCGTACTG |  |
